# Comparison of Dietary Intake between Polycystic Ovary Syndrome Women and Controls

**DOI:** 10.5539/gjhs.v8n9p302

**Published:** 2016-01-31

**Authors:** Farnaz Shishehgar, Fahimeh Ramezani Tehrani, Parvin Mirmiran, Sepideh Hajian, Ahmad Reza Baghestani, Nazanin Moslehi

**Affiliations:** 1 Department of Midwifery & Reproductive Health, School of Nursing & Midwifery, Shahid Beheshti University of Medical Sciences, Tehran, Iran; 2 Reproductive Endocrinology Research Center, Research Institute for Endocrine Sciences, Shahid Beheshti University of Medical Sciences, Tehran, Iran; 3 Nutrition and Endocrine Research Center, Research Institute for Endocrine Sciences, Shahid Beheshti University of Medical Sciences, Tehran, Iran; 4 Department of Clinical Nutrition and Dietetics, Faculty of Nutrition Sciences and Food Technology, National Nutrition and Food Technology research Institute, Shahid Beheshti University of Medical Sciences, Tehran, Iran; 5 Department of Biostatistics, School of Paramedical Sciences, Shahid Beheshti University of Medical Sciences, Tehran, Iran

**Keywords:** case control study, dietary intake, Polycystic Ovary syndrome

## Abstract

Polycystic Ovary syndrome (PCOS) is a complicated endocrinopathy affecting women in reproductive age. The crucial role of obesity and insulin resistance in progression of metabolic and cardiovascular features of PCOS has been confirmed. Although it has been suggested that there is a possible association between dietary pattern and risk of PCOS, few studies investigating the diet composition of PCOS women. The aim of this study was to compare the dietary intakes between women with polycystic ovary syndrome (PCOS) and eumenorrheic non hirsute women. This was a case control study of 142 women with PCOS and 140 eumenorrheic non hirsute healthy age and BMI matched controls. We compared the dietary intakes of our study group using a validated food frequency questionnaire (FFQ), using T-test or Mann-Whitney to compare the means of two groups. One way Anova was used to compare the tertiles of GI and GL in each group and a two way ANOVA was used to compare between tertiles of GI-GL and groups. The results demonstrated that energy and macronutrient intakes in PCOS women compared to controls were similar. PCOS group consumed more food items with high glycemic index (*p*=0.042) and less legumes (*P*=0.026) and vegetables (*p*=0.037) than controls. Both groups in the highest tertile of glycemic load (GL) had higher body mass index and waist circumference. Considering the results of this study, it was concluded that PCOS women had a dietary pattern that was characterized by a higher consumption of high GI food items and lower legumes and vegetables.

## 1. Introduction

Polycystic Ovary syndrome (PCOS) is a complicated endocrinopathy affecting women of reproductive age. According to the difference in the diagnostic criteria and ethnic back ground, the prevalence of this syndrome is reported to range between 2.2%–26% (March et al., 2010). In Iran, based on National Institute of Health (NIH) criteria, the prevalence of PCOS is reported 7.1% and according to Rotterdam criteria is 14.6% (Ramezani Tehrani et al., 2011). The short and long term consequences of this disorder, in addition to its impacts on general and mental health, impose heavy financial burdens on society (Azziz, Marin, Hoq, Badamgarav & Song, 2005). Previous studies reported a higher incidence of impaired glucose tolerance, type 2 diabetes and hyperinsulinemia, insulin resistance and obesity in PCOS women. Given the wide range of PCOS symptoms and its adverse consequences, several medical treatments, based on the complications and symptoms, have been prescribed (Moran, & Teede, 2009; Javanmanesh, Kashanian, Rahimi, & Sheikhansari, 2015). The goals of treatment are weight loss, improved hormonal and reproductive function, prevention of metabolic disorders and improved quality of life (Moran, Hutchison, Norman, & Teede, 2011). Also, the high cost of treatments and consequent complications have motivated researchers to identify the modifiable risk factors associated with this syndrome (Chavarro, Rich-Edwards, Rosner, & Willett, 2007). Although more than 50% of PCOS women are obese, there is limited data investigating the diet composition of PCOS women (Douglas, Norris, Oster, Darnell, Azziz, & Gower, 2006) documented inconsistent results from studies mostly conducted in western countries. Some studies report no difference in macronutrient and energy intake in women with and without PCOS (Douglas et al., 2006; Wright, Zborowski, Talbott, McHugh-Pemu, & Youk, 2004). In a recent study, higher intakes of fiber and micronutrients and lower intakes of food with a high glycemic index (GI) and saturated fatty acid have been reported in PCOS women as compared to controls (Moran, Ranasinha, Zoungas, McNaughton, Brown, & Teede, 2013). Furthermore, studies comparing dietary intake in PCOS women with controls, have reported higher intakes of saturated fat, and GI diet and lower intakes of fiber (Douglas et al., 2006; Colombo et al, 2009). Some cohort studies, documented increased risk of ovarian infertility to be associated with protein intakes from animal sources, carbohydrate with high GI (Chavarro et al., 2007, 2008, 2009). Since food habits are rooted in the culture of each region of the world (Cassel, 1957) and the relationship between dietary habits and incidence of some cardiovascular disease and type 2 diabetes has been demonstrated, it is essential to compare dietary habits of PCOS women with age-BMI matched controls to design appropriate dietary intervention. The aim of this study is to compare the dietary intakes (macronutrients, glycemic Index, glycemic load) between PCOS women and controls.

## 2. Materials and Methods

### 2.1 Study Design

This case control study included 142 PCOS women and 140 controls matched for BMI and age. The case group (N=142) were recruited from PCOS women aged between 18-40 years, attending the reproductive endocrinology research center, Shahid Beheshti University of Medical Sciences from January to July 2014. PCOS was defined using Androgen Excess Society (AES) for PCOS as the combination of menstrual dysfunction and clinical hyperandrogenism and/or hyperandrogenemia (HA), after excluding hyperprolactinemia, thyroid dysfunction, nonclassic 21-hydroxylase deficiency (NCCAH) and Cushing’s syndrome (Azziz et al., 2006). Hirsutism was assessed by Modified Ferriman-Gallwey and score> 8 is defined as hirsutism (Hatch, Rosenfield, Kim, & Tredway, 1981). Healthy controls were 140 age and BMI matched with PCOS women, who were attending to the clinics for annual gynecologic examinations. The exclusion criteria for both groups were being pregnant or on lactation, chronic disease (liver, kidney disease, hypertention, hyperprolactinemia, hypo or hyperthyroidism, diabetes), using of insulin or sensitizing agents, contraceptive pills, any medication affecting appetite or weight, special diet or exercise for weight loss, or having any history of malignancy. Sample size was defined using formula with CI 95% as 140 women in each group.


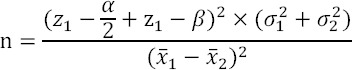


### 2.2 Measurement Instrument

A comprehensive questionnaire consisting demographic, reproductive, menstrual history and food frequency questionnaire, was filled during face to face interview by the main investigator. The study was approved by the ethical committee of research institute for endocrine science, Shahid Beheshti Medical sciences, and all participants signed a written informed consent form.

#### 2.2.1 Anthropometric Assessment

Height was measured to the nearest 1 cm, without shoes, in a standing position by using a portable height meter measuring device. Weight was measured with a digital scale with an accuracy of 100 gram. After each 5 weight measurements, the digital scale (Seca, Hamburg, Germany) was calibrated with standard weight control. BMI was calculated by dividing weight in kilograms to square of height in meters. According to WHO protocol, participants were classified as follows: BMI 18-24.9 as normal, BMI 25-29.9 as overweight and BMI > 30 as obese (Gill, Antipatis, & James, 1999). Waist was measured with tape at narrowest point between the last rib and pelvic crest, at the end of exhalation. Hip circumference was measured at the widest part then waist to hip ratio was calculated (Lohman, Roche, & Martorell, 1988).

#### 2.2.2 Dietary Assessment

Dietary intake was assessed by a valid semi quantitative food frequency questionnaire (FFQ) (Mirmiran, Esfahani, Mehrabi, Hedayati & Azizi, 2010) during face to face interviews. The FFQ includes 147 food items with a standard portion size. Participants were asked to report the frequency of consumption of each food item per day, week or month during the previous year. We used a food photo album that illustrated 3-4 different portion sizes to remind participants of how much food they had been consumed. The reported amount of each food item was converted to grams using household measures (Ghafarpour, Houshiar-Rad, & Kianfar, 1999). Energy and nutrient intake of each food item was calculated by multiplying the frequency of consumption of each food item by the nutrient content of the specified portion. The nutrient content of each food item was determined using US department of agriculture’s food composition table (USDA) because the Iranian food composition table is incomplete. For mixed meals, nutrients were calculated based on their components. GI was calculated by the International table of GI (Atkinson, Foster-Powell, & Brand-Miller, 2008) using glucose as a reference food. However, Iranian GI table (Taleban, & Esmaeili, 1999) was used for some food items not listed in the international table of GI. The GL of a serving of each food item was calculated by multiplying the carbohydrate content of each food per serving by the food’s GI value, and that product was divided by 100. The GL for each food item was then multiplied by the daily frequency of intake of each food item, and these values were summed over all food items to calculate total GL. Dietary GI was determined by dividing total dietary GL by total daily carbohydrate intake and multiplying value by 100. All participants were classified based on tertiles of GI and GL. Food items with similar nutritional features were pooled into 15 food categories which were as follows: High GI food items, moderate and low GI food items, low fat dairy, high fat dairy, legumes, vegetables, starchy vegetables, fruits, carbonated drinks, red meat, chicken, fish, egg, fast food, oils. High GI food items (GI >70) included boiled potatoes, white bread, biscuits, cake, baked fava beans. We estimated basal metabolic rate (BMR) from the standard equations based on age, sex and weight (Commission of the European Communities, 1992). Then for each participants energy intake/BMR was calculated and individuals with energy intake/basal metabolic rate above 2.4 (over reporters) or lower than 1.34 (under reporters) were excluded (Goldberg et al., 1991).

#### 2.2.3 Statistical Analysis

All statistical analysis was carried out using SPSS (version 20) and values < 0.05 were considered statistically significant. For data with normal distribution, student T test was used to identify differences between two groups and Mann Whitney test was used for data that were not normally distributed. Chi square test was used for categorical data. Macronutrient intakes, BMI, and Waist circumference according to tertiles of GI and GL were examined using One way Anova in each group and two way ANOVA was used to compare between tertiles of GI-GL and groups.

## 3. Result

Characteristics of the study population are presented in Table 1. PCOS women had higher weight and hip circumference, but lower waist/hip ratio compared to controls.

**Table 1:** The characteristics of women with polycystic ovary syndrome compared to controls

Factors	Case	Control	Test
Age(years)	28.56±4.86	28.95±5.78	P=0.631
Weight(kg)	69.37±14.97	67.45±12.92	P=0.048
BMI(kg/m^2^)	26.56±5.67	26.04±4.75	P=0.133
Waist circumference (cm)	85.26±13.96	85.16±12.89	P=0.893
Hip circumference (cm)	104.9±10.1	101.5±10.73	P=0.007
Waist to hip ratio(cm)	0.80±0.73	0.83±0.09	P=0.031
Pregnancy status			
Never pregnant	124(87.3%)	72(51.4%)	P<0.001
Ever pregnant	18(12.7%)	68(48.6%)	
Menstrual Intervals	40(30-60)	30(30-31)	P<0.001
Hirsutism score (The median and interquartile range)	6(3-10)	0(0-2)	P<0.001

Data are presented as mean ± SD, median (25^th^-75^th^ percentiles), and number (%).

Energy and macronutrient intakes in PCOS women compared to controls were similar, but the proportion of energy intake from polyunsaturated fatty acid (PUFA) was significantly higher in women with PCOS, compared to controls. There was no difference in GI and GL intake in women with or without PCOS. PCOS women, compared to controls had significantly higher intakes of sodium (*P*=0.01) (Table 2).

**Table 2:** Dietary intakes of PCOS and control groups

Variables	Case(n=142)	Control(n=140)	Test
Energy(K cal)	2457.8±572.7	2502.5±519.3	P=0.49
Carbohydrate(gram/day)	344.30±86.6	355.3±80.29	P=0.27
Carbohydrate (%)	56.19±6.83	56.93±6.46	P=0.34
Protein(gram/day)	66.15±23.05	70.68±41.96	P=0.26
Protein (%)	10.77±2.63	11.24±4.7	P=0.30
Fat(gram/day)	90.66±30.77	88.72±27.12	P=0.57
Fat (%)	33.03±7.04	31.82±6.56	P=0.13
Saturated fat(gram/day)	26.63±9.93	27.33±8.27	P=0.52
Saturated fat (%)	9.71±2.63	9.86±2.63	P=0.52
MUFA^1^(gram)	27.48±10.24	26.05±9.40	P=0.22
MUFA(%)	10.04±2.74	10.30±2.68	P=0.24
PUFA^2^(gram)	20.38±9.7	18.91±8.42	P=0.17
PUFA(%)	7.42±2.63	6.77±2.63	P=0.042
Fiber(gram/day)	19.04(12.14-29.60)	21.39(14.7-34.38)	P=0.12
Sodium(mg/day)	4778.66±1394.36	3894.56±1063.4	P=0.01
Glycemic index	61.22±6.26	60.54±5.51	P=0.32
Glycemic load	166.61(132.52-194.15)	155.01(130.07-185.69)	P=0.38

Data are presented as mean ± SDs and median (25^th^-75^th^ percentiles).

1-Monounsaturated fatty acid; 2- Poly unsaturated fatty acids.

Classification of dietary and anthropometric factors based on tertiles of GI and GL are presented in Table 3, illustrating that, in both groups, those in the third tertile, compared to those in the first tertile, had higher BMI and waist circumference, however in the GI tertiles, this differences was not statistically significant. PCOS women in the third tertile of GL, compared with those in the first tertile, had significantly higher BMI and waist circumference (*P*<0.001).

**Table 3:** Dietary and anthropometric factors in women with Polycystic Ovary syndrome and controls classified by tertiles of Glycemic Index and Glycemic load

Variables	1^st^ tertile(n=47) GI Case	2^nd^ tertile(n=48) GI Case	3^rd^ tertile(n=47) GI Case	P value	1^st^ tertile(n=46) GI control	2^nd^ tertile(n=47) GI control	3^rd^ tertile(n=47) GI control	P value	P value
Carbohydrate (%)	56.32±6.65	54.59±6.90	57.62±6.73	P=0.095	58.12±7.52	56.91±4.92	55.79±6.63	P=0.223	P=0.098†
Protein (%)	11.05±2.72	9.88 ± 2.55	11.36±2.43^b,c^	P=0.016	11.41±1.91	11.32±2.44	10.99±7.57	P=0.9	P=0.369†
Fat (%)	32.61±6.66	35.51±7.54^b,c^	31 ±6.23	P=0.006	30.45±7.78	31.76±4.91	33.21±6.56	P=0.129	P=0.005†
BMI	25.69±4.75	25.97±5.36	28.04±4.75	P=0.081	25.89±5.17	25.17±4.65	25.26±4.48	P=0.732	P=0.185†
Waist circumference	82.68±11.41	84.22±13.79	88.91±11.24	P=0.07	86.66±14.42	83.42±11.03	85.08±13.03	P=0.482	P=0.144†

Variables	1^st^ tertile(n=47) GL Case	2^nd^ tertile(n=48) GL Case	3^rd^ tertile(n=47) GL Case	P value	1^st^ tertile(n=46) GL control	2^nd^ tertile(n=47) GL control	3^rd^ tertile(n=47) GL control	P value	P value

Carbohydrate(%)	53.15±6.90	56.56±6.38	58.85±6.07^a,c^	P<0.001	53.51±7.63^a,b^	57.7±5.54	59.51±4.40^a,c^	P<0.001	P<0.001†
Protein (%)	11.13±2.8	10.81±2.49	10.37±2.59	P=0.37	12.7±7.4	10.7±2.48	10.35±1.96^a,c^	P=0.033	P=0.023†
Fat (%)	35.71±7.34	32.62±6.01	30.77±6.89^a,c^	P<0.002	33.78±7.90^a,c^	31.59±6.31	30.13±4.75	P=0.025	P=0.001†
BMI	24.77±4.06	25.76±7.03^b,c^	29.17±5.67^a,c,*^	P<0.001	24.12±3.52	25.70±4.89	26.46±5.41^a,c,*^	P=0.05	P=0.001†
Waist circumference	81.10±9.4	82.77±12.05^b,c^	92.89±7.11^a,c,*^	P<0.001	81.39±10.16	86.59±9.29	87.12±8.24^a,c, *^	P=0.047	P<0.001†

In the case group 1^st^ tertile GI 57.78, 2^nd^ tertile GI> 57.78 and <64.25, 3^rd^ tertile GI >64.25 In control group, 1^st^ tertile GI <57.9, 2^nd^ tertile GI >57.9 and <62.84, 3^rd^ tertile GI 62.84. †For multiple comparison a two way anova was used. ^a,b,c^ Different letters showed significant difference among tertiles in the same group (P<.05).

In the case group 1^st^ tertile GL <141.12, 2^nd^ tertile GL> 141.12 and <168.67, 3^rd^ tertile GL >168.67. In control group, 1^st^ tertile GL≤ 142.54, 2^nd^ tertile GL > 142.54 and < 182.26, 3^rd^ tertile GL ≥ 182.26. † For multiple comparison two way anova was used. Different letters showed significant difference among tertiles in the same group (P<.05). Asterisk showed significant difference among tertiles in cases and controls.

PCOS women had significantly higher consumption of high GI food items and egg than those without PCOS. Vegetable and legume intakes were significantly lower in cases than controls (*P*<0.05) and the consumption of high fat dairy in cases was lower than controls but the difference was not statistically significant (*P*= 0.065) (Table 4).

**Table 4:** Food group intakes in PCOS women and controls

Food groups	Case(n=142)	Control(n=140)	Test
High GI food items(g/day)	95(62.63-159.46)	89.86(45.38-137.63)	P=0.042
Medium and low GI food items(g/day)	366(272.72-536.07)	414.67(297.24-514.32)	P=0.355
Low fat dairy(g/day)	206.42(54.76-351.83)	187.03(101.10-327.19)	P=0.530
High fat dairy(g/day)	73.06(13.77-233.41)	108.23(33.11-240)	P=0.065
Legumes (g/day)	19.86(9.53-35.61)	24.38(14.64-40.31)	P=0.026
Vegetables (g/day)	284.74±157.38	323.68±147.31	P=0.037
Starchy vegetables(g/day)	24.05(13.12-37.39)	22.27(13.53-35)	P=0.590
Fruits(g/day)	386.73±211.08	388.89±188	P=0.922
Carbonated drinks(g/day)	13.33(1.64-57.14)	16.66(1.64-57.14)	P=0.731
Red meat (g/day)	19.82(12.16-29.39)	20.64(12.34-31.58)	P=0.906
Chicken(g/day)	24.28(12.14-36.42)	23.28(12.14-35.42)	P=0.597
Fish(g/day)	6.74(3-13.82)	6.58(3.3-14.45)	P=0.927
Egg(g/day)	22.65±16.98	17.74±12.99	P=0.031
Fast foods(g/day)	10.53(4.02-25.18)	11.79(3.75-27.49)	P=0.940
Oils(g/day)	6(4.21-12)	6(3.42-12)	P=0.323

High GI food items, boiled potatoes, white bread, biscuits, cake, baked fava beans;

Low-fat dairy, low-fat milk and yoghurt (<2% of total fat content), and low-fat cheese (<20% of total fat content);

High fat dairy, high-fat milk, high-fat yoghurt, high-fat cheese, and chocolate milk;

Legumes, All kind of legumes including beans, peas, lima beans, lentils, soy;

Vegetable, Green leafy vegetables (Spinach, lettuce, mixed vegetables), Cruciferous vegetables, tomatoes, carrots, cucumbers, eggplants, corn, garlic, turnips, squash, mushrooms, onions, pumpkin, celery, green peas, green beans, green paper, potatoes;

Starchy vegetable, potato, pumpkin, green peas, fava beans.

Red-meat, Beef, lamb;

Fish, Canned tuna fish, other fish;

Fast food, Pizza, processed meats, hamburger, French fries;

Oils, Vegetable oils and olive oil.

## 4. Discussion

Our result shows no difference in energy or macronutrient intakes among PCOS women and healthy controls. However PCOS women had higher consumption of high GI food items and lower intakes of vegetables and legumes than their controls. Percentage of energy from macronutrient intake was within the acceptable macronutrient dietary intake (AMDR) in both groups, findings is consistent with those of Wright et al. (2004) and Douglas et al. (2006), Altieri et al. (2013), Toscani et al. (2011), all of whom reported no difference in energy and macronutrient intakes in women with PCOS compared to non PCOS women. In our study, the percent of energy from fat was higher than controls, difference not statistically significant (*P*=0.13), results similar to those of Douglas et al. (2006) who reported PCOS women consumed more fat, difference not significant (69.2± 25 versus 61.5± 21.1 g/day, P=0.22). The energy intake(%) from fat in our case group are contrary to those of Altieri et al. (2013) who reported a lower percent of energy intake from fat in PCOS women, an inconsistency which may be due to differences in study population, as their study population included overweight or obese women. Furthermore, in different parts of the world there are various nutritional habits that affect nutritional choices. Our data showed the percent of energy intake from saturated fat was comparable to controls. Unlike our findings, other studies showed that PCOS women consumed more fat (Ahmadi, Akbarzadeh, Mohammadi, Akbari & Tolide, 2013) and saturated fat (Wild, Painter, Coulson, Carruth & Ranney, 1985) than controls, a difference which may be related to participants’ weight; in latter study, PCOS women were heavier than controls. Furthermore, all of those studies used 3 or 4 day 24 hour dietary recall questionnaires, for which respondents were required to be highly motivated to complete; hence the individuals who participate to record food intake, may be limited and the generalizability of those findings to population may be confined. Our results showed fiber intakes were similar in both groups (*P*=0.12) and were consistent with Adequate Intake (AI) in women aged 19-50 years (25 gram per day). These results are similar to those of studies performed by Douglas et al. (2006) and Toscani et al. (2011) but in contrast to those of Moran et al. (2013) who showed that PCOS women consumed more fiber than controls. Given the documented role of fiber in improving insulin sensitivity and lowering cholesterol, the results of those studies demonstrating higher fiber intake in PCOS women, compared with controls should be interpreted with caution. There is no difference in GI and GL intake in PCOS women and age-BMI matched controls. These results are in agreement with those reporting no difference in GI intakes of women with or without PCOS (Douglas et al., 2006; Graff, Mário, Alves, & Spritzer, 2013). However our data are contrary to those of Moran who showed in PCOS women GI intake was lower than in controls; this controversy may be due to the selection of their study population, in that study, the diagnosis of PCOS women, based on self reporting. Therefore, the case group may not be representative of women with PCOS. There is no difference in BMI and waist circumference based on classification of study population by tertilies of GI. Our results are not in agreement with those of Graff et al. (2013) who revealed women with or without PCOS in the third tertile of GI had higher BMI and waist circumference than those in the first tertile, a controversy which may be due to the differences in culture and food habits of study population. Grains form the staple diet in Iran and variety of food items made from whole grain is limited. So selection of different types of grain is limited and refined grain makes most to total carbohydrate intake leading to increased GI intake. Our results in respect to classification of study population based on GL tertiles are remarkable. Those with higher GL (third tertile), had higher BMI and waist circumference than those in the first tertile (Table 3). In PCOS women, the relationship between third tertile of GL and BMI and waist circumference was greater than controls. High GI or GL diets led to higher blood glucose levels and insulin response, decreasing resting energy expenditure (Pereira et al., 2004), the diets also increased fat accumulation (Augustin, Franceschi, Jenkins, Kendall, & Vecchia, 2002). There is controversial data on the association of dietary GI, GL with BMI and waist circumference (Mosdol, Witte, Frost, Marmot & Brunner, 2007). Some evidence indicates inverse or no relationship (Gaesser, 2007; Mendez, Covas, Marrugat, Vila, & Schroder, 2009), while a positive relationship has also been documented in other studies (Murakami et al., 2007).

Given the fact that GI could estimate the quality of carbohydrate and GL measures the GI and dietary carbohydrate of each food item, GL could represent both quality and quantity of carbohydrate consumed. Previous studies have documented the physiologic creditability of dietary GL to predict postprandial glycemia and insulinemia (Liu et al., 2000). In nutritional studies, the relative risk of chronic disease is related to dietary GL which has been proven to be more appropriate for predicting glycemia and insulinemia than GI (Bao, Atkinson, Petocz, Willett & Brand-Miller, 2011). In our study, compared to controls, PCOS women consumed more food items with high GI (*P*=0.042), results are consistent with those of Altieri et al. (2013) and Douglas et al. (2006) who demonstrated higher consumption of starchy food with high GI in PCOS women. Higher consumption of GI food has also been related to a greater risk of ovulatory infertility. High carbohydrate (CHO) diet leads to hyperinsulinemia and reduced sex hormone binding globulin (SHBG) (Chavarro et al., 2009). It has been believed that insulin sensitivity is a key factor in ovarian function and fertility (Chavarro et al., 2008). Both quality and quantity of CHO in diet affects glucose metabolism and insulin sensitivity in PCOS and healthy women (Mendez et al., 2009). The results of this study showed lower consumption of legumes in PCOS women compared to controls (*P*=0.026). There is evidence suggesting that consumption of animal protein is associated with higher chance of ovulatory infertility. In addition, increasing vegetable protein intake resulted in improving insulin sensitivity and increased rates of ovulation. Since insulin sensitivity and glucose homeostasis affect reproductive function and fertility, different sources of protein with different effects on glucose homeostasis and insulin sensitivity could also have the same effect (Chavarro et al., 2008). Our results showed vegetable intake in PCOS women significantly lower than controls (*P*=0.037), a finding not consistent with that of Altieri et al. (2013) who showed no difference in vegetable intake in two groups, the inconsistency may be due to study population and tools for evaluating food intake. Neither are our results on oil intake in line with those of Altieri et al. (2013) who reported higher intake of oil (especially olive oil) in PCOS women compared with controls.

Given the known role of olive oil on carbohydrate and lipid metabolism and oxidative stress, in this respect, it was suggested that more studies are needed. In respect to consumption of other food categories, our data showed that the two groups were comparable. PCOS women had significantly higher intake of sodium than controls (*P*<0.05). Regarding sodium intakes our results are consistent with those of Graff et al. (2013) who showed higher sodium intake in PCOS women compared with controls. Mean sodium intake in both groups was higher than AI (1500 mg per day) and upper limit (2300 mg per day). Diminishing salt intake to less than 5.0 g/day has been recommended by a joint WHO/FAO working group (2003). In Iran, the mean daily salt intake was reported 10±4.8 gram per day in males and 7.5±3.3 gram/day in females (Mirzaei, Mohammadhossien, Namayandeh, & Gharahi Ghehi, 2014). The results of a cohort study determined, high sodium intake leads to elevated glucocorticoid production in fatty tissue and urine cortisol metabolites (Baudrand et al., 2014). In addition, high sodium intakes were reported to be associated with homeostatic model assessment and hyperglyceridemia and lower adiponectin levels (Vedovato et al., 2004).

The main strength of our study is its design to select diagnosed PCOS women according to AES criteria and compare the dietary intake of them with age and BMI matched controls. Furthermore, we used a valid FFQ for evaluating dietary habits, which is a validated method used to evaluate long term dietary habits in case-control or cohort studies to examine the relationship between dietary intake and disease risk (Sempos, Liu, & Ernst, 1999). The validity of our study is increased by defining and omitting all potential confounders. In most dietary surveys, there is a risk for under and over reporting food intakes as nutritional data is collected through participants’ self reports based on their memory; however in analysis, we excluded under and over reporters. The weakness of this study is that the associations of metabolic and hormonal characteristics of study population with food categories weren’t examined. The nature of our case control design hinders our ability to infer causality between dietary intake and PCOS status.

## 5. Conclusion

The results of this study suggested that energy and macronutrient intakes in PCOS women did not differ from age-BMI matched controls. Although caloric intakes in both groups were the same, PCOS women consumed more food items with high GI and sodium than controls. PCOS women compared to controls, consumed a lower quantity of vegetable and legumes. Further research is warranted to define the effectiveness of low GI and GL diets on metabolic and reproductive outcomes in PCOS women.
